# Two PTP receptors mediate CSPG inhibition by convergent and divergent signaling pathways in neurons

**DOI:** 10.1038/srep37152

**Published:** 2016-11-16

**Authors:** Yosuke Ohtake, Daniella Wong, P. M. Abdul-Muneer, Michael E. Selzer, Shuxin Li

**Affiliations:** 1 Shriners Hospitals Pediatric Research Center, Lewis Katz School of Medicine at Temple University, Philadelphia, PA 19140, USA; 2 Department of Anatomy and Cell Biology, Lewis Katz School of Medicine at Temple University, Philadelphia, PA 19140, USA; 3 Department of Neurology, Lewis Katz School of Medicine at Temple University, Philadelphia, PA 19140, USA

## Abstract

Receptor protein tyrosine phosphatase σ (PTPσ) and its subfamily member LAR act as transmembrane receptors that mediate growth inhibition of chondroitin sulfate proteoglycans (CSPGs). Inhibition of either receptor increases axon growth into and beyond scar tissues after CNS injury. However, it is unclear why neurons express two similar CSPG receptors, nor whether they use the same or different intracellular pathways. We have now studied the signaling pathways of these two receptors using N2A cells and primary neurons derived from knockout mice. We demonstrate that both receptors share certain signaling pathways (RhoA, Akt and Erk), but also use distinct signals to mediate CSPG actions. Activation of PTPσ by CSPGs selectively inactivated CRMP2, APC, S6 kinase and CREB. By contrast LAR activation inactivated PKCζ, cofilin and LKB1. For the first time, we propose a model of the signaling pathways downstream of these two CSPG receptors. We also demonstrate that deleting both receptors exhibits additive enhancement of axon growth in adult neuronal cultures *in vitro*. Our findings elucidate the novel downstream pathways of CSPGs and suggest potential synergy of blocking their two PTP receptors.

After CNS injuries, a family of extracellular matrix (ECM) molecules, the chondroitin sulfate proteoglycans (CSPGs), are highly upregulated by reactive scars and potently inhibit axon growth into and beyond the lesion area^1^,^2^,^3^. CSPGs may be attached to cell membranes and usually form perineuronal nets by linking with several elements of the ECM, including hyaluronan, tenascin R and other molecules. The inhibitory effect of CSPGs on axon extension has been known for over 25 years^4^,^5^, but the underlying molecular mechanisms are not well understood^6^,^7^. CSPGs consist of glycosaminoglycan (GAG) chains attached to core proteins, and both components exert inhibition on neurite outgrowth^8^.

The presence of sulfated GAG chains is particularly important because removing GAGs or preventing GAG sulfation neutralizes most of the suppression of axon growth by CSPGs *in vitro*^9^,^10^,^11^. Although CSPGs may block growth by sterically hindering growth-promoting adhesion molecules (such as laminins/integrins) and facilitating inhibition by chemo-repulsive molecules, such as Sema 3a and 5a^12^,^13^,^14^, several receptors appear important in conveying CSPG inhibition, including PTPσ, LAR, and Nogo receptors (NgR) 1 and 3^15^,^16^,^17^,^18^,^19^. In particular, two members of the LAR subfamily, PTPσ and LAR, bind CSPGs with high affinity and mediate their suppression of axon elongation.

Deficiency of PTPσ or LAR in adult mice increased regrowth of various projection tracts after spinal cord injury (SCI), including sensory, corticospinal and serotonergic axons, into the caudal spinal cord^15^,^17^,^18^. Pharmacological blockade of LAR or PTPσ with small peptides stimulated regrowth of serotonergic axons and functional recovery after transection or contusion SCI^16^,^19^. However, the downstream signals mediating the effects of these two similar receptor-type PTPs (RPTPs) and the molecular basis for their requirement by neurons are not known. Nor is it known whether blocking both receptors would exert identical or synergistic therapeutic actions. We have now studied the intracellular signaling pathways that mediate CSPG-LAR/PTPσ interactions and compared their effects on the activities of multiple signaling proteins in cultures of a neuronal cell line, N2A. We further confirmed our findings in N2A cells with primary cultures of postnatal cerebellar neurons derived from PTPσ or LAR knockout mice. We demonstrate that LAR and PTPσ employ some signaling pathways in common, including RhoA, Akt, extracellular-signal-regulated kinase (Erk) and microtubule-associated-protein 1B (MAP1B). Their actions in transmitting CSPG effects to inhibit axon growth also involve distinct signals, including the use by PTPσ of collapsin response mediator protein 2 (CRMP2), adenomatous polyposis coli (APC), S6 ribosomal protein (S6) and cAMP response element-binding protein (CREB), and the use by LAR of cofilin, protein kinase C ζ (PKCζ) and liver kinase B1 (LKB1). Consistent with this, deletion of both receptors showed additive enhancement of axon growth in adult neurons *in vitro*.

This study supports a novel model of signaling pathways downstream of the two CSPG receptors and potential synergistic actions of blocking both simultaneously.

## Results

### RhoA signaling mediates actions of both receptors, LAR and PTPσ

RhoA is a prominent intracellular signal that regulates cytoskeletal dynamics, transcription and cell growth, and is thought to mediate functions of multiple axon growth inhibitors, including myelin- associated inhibitors^20^, CSPGs^21^,^22^ and guidance cues Sema 3a and Ephrin^23^,^24^. Inhibition of RhoA by C3 and the non-steroidal anti-inflammatory drugs ibuprofen and indomethacin, or of its downstream Rho kinase (Rock) by Y27632, Fasudil and dimethylfasudil, overcame CSPG-triggered inhibition and promoted neurite outgrowth in various cell types, including dorsal root ganglion (DRG) neurons^21^, Ntera-2 cells^25^ and retina ganglion cells^22^. To test whether RhoA mediates the actions of PTPσ or LAR due to CSPG stimulation, we transfected N2A cells with PTPσ or LAR and stimulated the cells with a mixture of purified CSPGs (neurocan, phosphacan, versican and aggrecan, 1.5 μg/ml) 48 hrs after transfection. Most N2A cells formed two or more neurites 2–3 days after culture (not shown), consistent with the neuronal character of this cell line^26^,^27^. We measured levels of active RhoA in cell lysates by precipitation with the rhotekin binding domain, which binds only the GTP-bound form of RhoA^28^,^29^.

**Figure 1:**
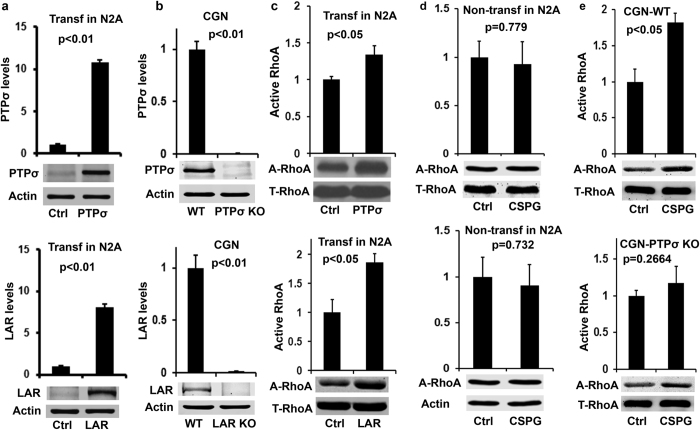
Effects of CSPG treatment on activities of RhoA in N2A cells transfected with LAR or PTPσ and in CGNs derived from postnatal PTPσ+/+ or PTPσ−/− mice. The levels of PTPσ or LAR protein were determined from lysates of N2A cells 48 hrs after transfection with control, PTPσ or LAR plasmid (**a**), or of CGN cultures (**b**) derived from PTPσ or LAR KO mice by Western blots. The levels of active RhoA were measured in lysates of N2A cells 48 hrs after PTPσ or LAR transfection or CGNs 24 hrs after cultures with pulldown assay (active RhoA) and Western blots. Application of a mixture of purified CSPGs (1.5 μg/ml, for 20 min) increased levels of active RhoA in both PTPσ and LAR (**c**) transfected cells and in PTPσ+/+, but not PTPσ−/−, CGNs (**e**). In contrast, CSPG stimulation in N2A cells without PTPσ or LAR transfection did not alter the levels of active RhoA (calibrated with either total RhoA or actin, (**d**). The full-length blots are included in the Supplementary Information file.

Treatment with CSPGs for 20 min significantly increased active RhoA levels in N2A cells transfected with either PTPσ or LAR, compared with those in control cells (Fig. 1c). Because CSPG treatment did not activate RhoA in non-transfected N2A cells (Fig. 1d), the overexpressed PTPσ or LAR must have been responsible for any RhoA activation induced by CSPGs. Expression of LAR appeared to induce slightly greater RhoA activation than did PTPσ (1.34 vs. 1.87). Thus, RhoA activation resulted from interactions between CSPGs and each of the RPTPs separately. To confirm expression of CSPG receptor proteins, we used Western blots to measure the levels of PTPσ and LAR in N2A cells 2 days after transfection. Control cells expressed low levels of both proteins, but transfection with either PTPσ or LAR dramatically elevated the levels of the corresponding protein - 11 fold for PTPσ and 8 fold for LAR (Fig. 1a).

To validate the findings from N2A cells in primary neuronal cultures, we evaluated whether PTPσ or LAR deletion alters activities of intracellular signals, including active RhoA, in cultured cerebellar granule neurons (CGNs) following incubation in CSPGs. We could easily collect enough samples for multiple Western blot analyses using CGN cultures derived from postnatal 7–10 day mice. Following 24 hours of growth, CGNs were treated with CSPGs (1.5 μg/ml) for 20 minutes and the levels of active RhoA in the supernatants were detected by precipitation with rhotekin binding domain beads and then by Western blotting. Application of CSPGs at 1.5 μg/ml significantly enhanced levels of active RhoA in CGNs derived from PTPσ+/+ mice (Fig. 1e). However, application of CSPGs (1.5 μg/ml) failed to induce significant changes in active RhoA in cultured CGNs derived from PTPσ−/− mice. Similarly, we demonstrated that CSPG stimulation activated RhoA in LAR+/+ CGNs, but not in LAR−/− neurons^16^. We confirmed the deficiency of PTPσ and LAR proteins in knockout CGNs (Fig. 1b).

**Figure 2:**
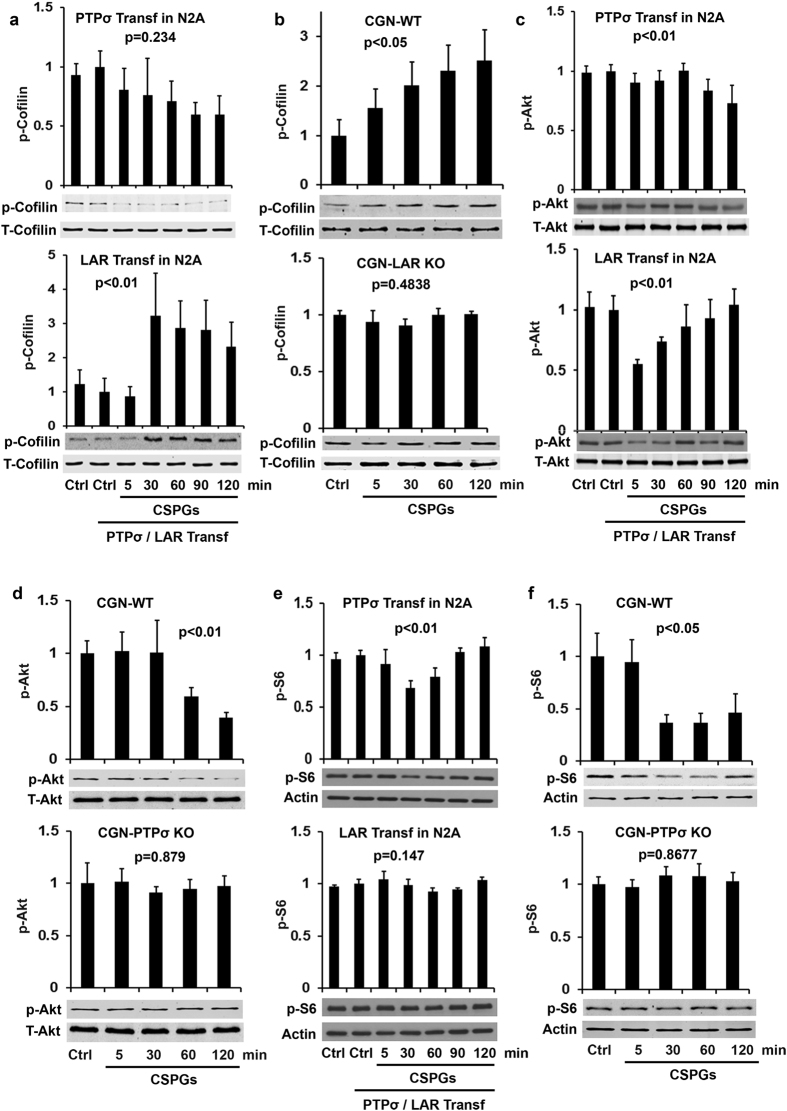
Effects of CSPG application on activities of cofilin, Akt and S6 kinase in N2A cells or CGNs. PTPσ or LAR transfected N2A cells or CGNs derived from PTPσ or LAR KO mice were treated with purified CSPGs (1.5 μg/ml) for different time points and the levels of phosphorylated Cofilin (p-Cofilin Ser3, inactive form), Akt (p-Akt s473, active form) and S6 kinase (p-S6 Ser235/236, active form) in cell lysates were measured by Western blots. CSPG stimulation enhanced levels of p-Cofilin in LAR transfected N2A cells (**a**) and in LAR+/+, not LAR−/−, CGNs (**b**). CSPG treatments decreased levels of p- Akt in both PTPσ and LAR transfected N2A cells (**c**) and in PTPσ+/+, not PTPσ−/−, CGNs (**d**). Also, CSPGs reduced levels of p-S6 in PTPσ, not LAR, transfected N2A cells (**e**), and in PTPσ+/+, not PTPσ−/− CGNs (**f**). The full-length blots are included in the Supplementary Information file.

As a downstream mediator of RhoA action, Rock can phosphorylate and activate LIM kinase (LIM-K), which in turn phosphorylates and inactivates cofilin^30^,^31^. As an actin binding protein, cofilin is critical to regulating actin dynamics, and phosphorylation of cofilin at Ser3 (inactive form) inhibits actin- depolymerizing activity at the minus end of filaments, thus preventing actin reassembly^32^,^33^. We examined levels of p-cofilin in N2A cells transfected with PTPσ or LAR and found dramatically elevated levels of p-cofilin in LAR-transfected but not PTPσ-transfected cells 30–120 min after incubation with CSPGs (Fig. 2a), suggesting that CSPG-LAR interactions increase cofilin phosphorylation and inhibit axon elongation by reducing actin reassembly. Consistently, Rock inhibitors attenuated p-cofilin levels in PC12 cells due to CSPG stimulation^34^. CSPG stimulation for 5–120 min also significantly enhanced the levels of p-cofilin in wild-type (WT) CGNs, but not in LAR−/− CGNs (Fig. 2b). Thus, inactivation of cofilin by phosphorylation appears important in mediating the inhibitory effects of CSPG-LAR, but not CSPG-PTPσ.

### Akt/mTOR pathway conveys signals by the two CSPG receptors

The intracellular PI3K/Akt pathway is essential for regulating neuronal growth during development and contributes to suppression of axon growth by CSPGs^16^,^21^,^22^,^35^. We evaluated whether Akt and its downstream signals mediate some actions of the two CSPG receptors in cultures. Phosphorylation of Akt at residue Ser473 in its C-terminal hydrophobic motif is necessary for its full activation, and cellular levels of p-Akt at Ser473 correlate with its activity^36^,^37^. CSPG stimulation reduced the levels of p-Akt in N2A cells transfected with either PTPσ or LAR (Fig. 2c). Consistently, application of CSPGs reduced levels of p-Akt in WT CGNs, but not in PTPσ−/− (Fig. 2d) or LAR−/−16 neurons. These results indicate that Akt mediates actions of both receptors upon CSPG application, although PTPσ activation inactivates Akt at later time points (60–120 vs. 5–30 min).

**Figure 3:**
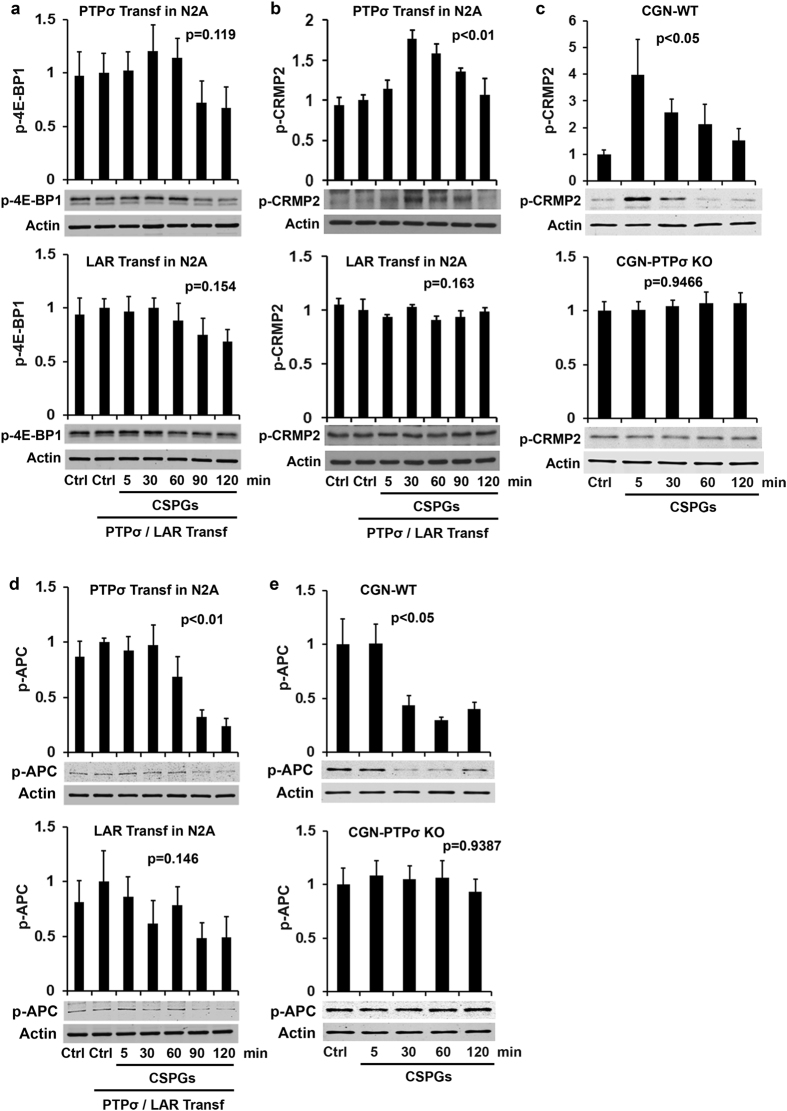
Effects of CSPGs on activities of 4E-BP1, CRMP2 and APC in N2A cells or CGNs. PTPσ or LAR transfected N2A cells or CGNs derived from PTPσ+/+ or PTPσ−/− CGNs were treated with purified CSPGs (1.5 μg/ml) at different time points and the levels of phosphorylated 4E-BP1 (p-4E-BP1 Thr37/46, inactive form), CRMP2 (p-CRMP2 Thr514, inactive form) and APC (p-APC Ser2054, active form) in supernatants of cell lysates were measured by Western blots. CSPG stimulation did not alter levels of p- 4E-BP1 in either PTPσ or LAR transfected cells significantly (**a**). CSPGs increased levels of p-CRMP2 in PTPσ, not LAR, transfected N2A cells (**b**), and in PTPσ+/+, not PTPσ−/−, CGNs (**c**). Also, CSPG applications attenuated levels of p-APC in PTPσ, not LAR, transfected N2A cells (**d**), and in PTPσ+/+, not PTPσ−/−, CGNs (**e**). The full-length blots are included in the Supplementary Information file.

Deletion of PTEN, a negative regulator of PI3K/Akt signaling, stimulated neuronal growth and survival following axotomy by activating mTOR^38^,^39^, a key signal regulating protein synthesis during cell growth. We examined whether mTOR pathways are modulated during CSPG-mediated inhibition by measuring p-S6 levels, which reflect mTOR activity^40^. Phosphorylation of the ribosomal protein S6, a downstream effector of mTOR, correlates well with increased translation of many mRNA transcripts and with cell growth. Overexpression of PTPσ, but not LAR, reduced levels of p-S6 in N2A cells after administration of CSPGs (Fig. 2e), suggesting that mTOR mediates the effects of the interactions between CSPGs and PTPσ, but not LAR. In support of this, CSPG stimulation attenuated levels of p-S6 in WT neurons, but not in PTPσ-deleted neurons (Fig. 2f). Of note, we compared alterations of several signaling proteins (RhoA, Cofilin, and Akt) calibrated with either total signaling proteins (Figs 1d and 2a–d) or actin (Supplemental Fig. 1), and obtained identical results with either control (see Supplemental Table 1). We thus employed actin as the loading protein for the most other signaling proteins studied here. mTOR regulates cell growth also by phosphorylation of the translation repressor protein 4E-BP1, which releases the eukaryotic initiation factor 4E (eIF4E) and initiates protein translation during growth. 4E-BP1 is primed for subsequent phosphorylation at Ser65 and Thr70 after its phosphorylation at Thr37/46 by FRAP/mTOR^41^. Therefore, we also measured the levels of p-4E-BP1 (Thr37/46, inactive), but found that overexpression of either PTPσ or LAR induced only a trend toward slight reduction in p-4E-BP1 levels in response to CSPG stimulation (Fig. 3a; not significant). This result indicates that the role of 4E-BP1 in regulating CSPG-receptor interactions is minimal.

CRMP2 is critical for regulating axon formation and outgrowth during development by binding tubulin heterodimers and promoting microtubule assembly^42^,^43^,^44^. Because activation of Rock and glycogen synthase 3β (GSK-3β), the downstream signal of Akt, contribute to axon growth cone collapse by phosphorylating and inactivating CRMP2^45^,^46^,^47^,^48^, we examined potential changes of p-CRMP2 at Thr514 (inactive), a target of GSK-3β. CSPG stimulation increased the levels of p-CRMP2 in PTPσ- but not LAR-transfected N2A cells (Fig. 3b), suggesting that phosphorylation of CRMP2 contributes to axon growth inhibition caused by CSPG-PTPσ interactions. Indeed, application of CSPGs increased levels of p-CRMP2 in WT CGNs, but had no effect in PTPσ-deleted neurons (Fig. 3c).

### APC and MAP1B mediate interactions between CSPGs and RPTPs

**Figure 4:**
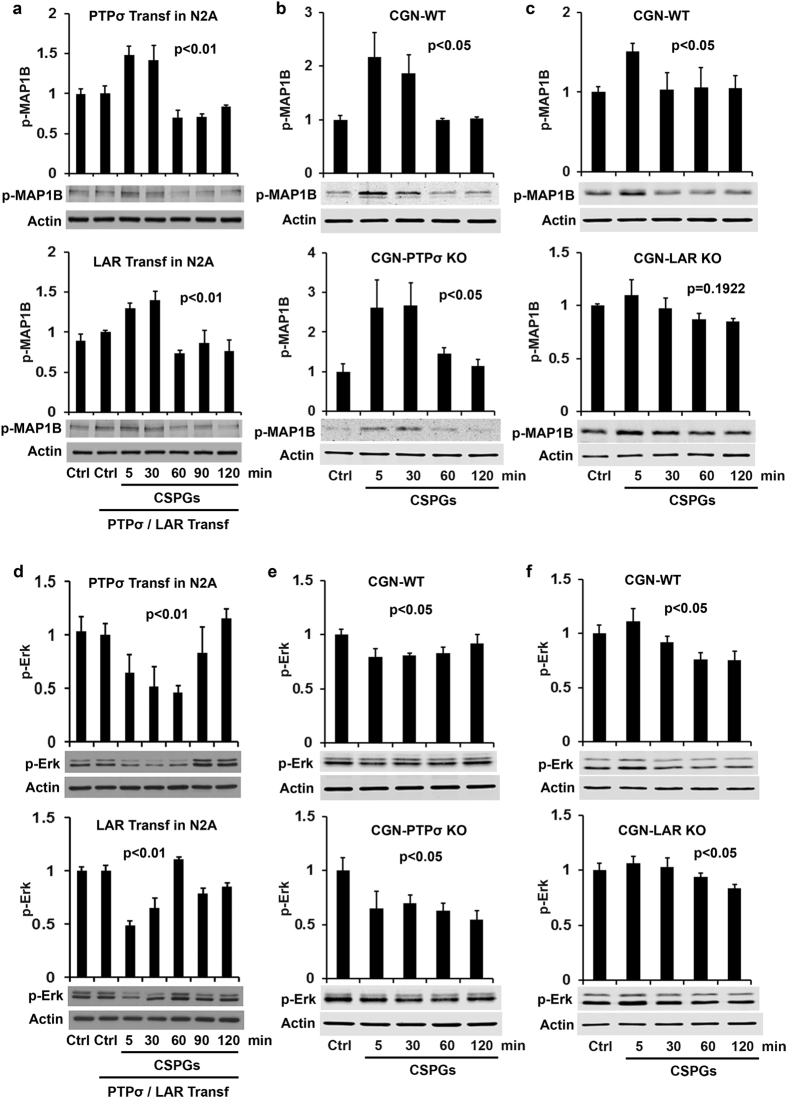
Effects of CSPGs on activities of MAP1B and Erk in N2A cells or CGNs. PTPσ/LAR transfected N2A cells or CGNs derived from PTPσ or LAR KO mice were treated with purified CSPGs (1.5 μg/ml) at several time points and the levels of phosphorylated MAP1B (p-MAP1B Thr1265, active form) and Erk (p-Erk1/2 p44/42 Ser2054, active form) in the supernatants of cell lysates were examined by Western blots. CSPG application enhanced levels of p-MAP1B in both PTPσ and LAR transfected N2A cells (**a**) and in WT CGNs (**b,c**). LAR, not PTPσ, deletion largely eliminated the enhanced p-MAP1B. Moreover, CSPGs attenuated levels of p-Erk in both PTPσ and LAR transfected cells (**d**) and in PTPσ+/+, PTPσ−/−, LAR+/+ and LAR−/− CGNs (**e,f**), suggesting that deleting one of RPTPs does not eradicate CSPG effects on Erk signal and that Erk conveys actions of both receptors. The full-length blots are included in the Supplementary Information file.

The tumor suppressor APC accumulates at the tip of the growth cone during axon elongation and its phosphorylation by GSK-3β suppresses axon growth by reducing its ability to bind microtubules^49^. Because Akt/GSK-3β signaling mediates CSPG function in PC12 cells and CNS neurons^16^,^21^,^35^, we evaluated the changes in p-APC levels after applying CSPG. This reduced p-APC levels in N2A cells transfected with PTPσ but not LAR (Fig. 3d). CSPGs similarly attenuated p-APC levels in WT CGNs, but not in PTPσ-deleted neurons (Fig. 3e). These data suggest that a reduction in APC levels is involved in mediating effects of CSPG-PTPσ interactions. As another well-known target of GSK-3β, MAP1B regulates microtubule dynamics in axon growth cones. In contrast to APC, phosphorylation by GSK-3β at Thr1265 of MAP1B activates this protein and reduces microtubule dynamics in axons. Treatment with CSPGs enhanced the levels of p-MAP1B in N2A cells overexpressing either PTPσ or LAR (Fig. 4a), suggesting that MAP1B mediates actions of both LAR and PTPσ. In support of this, levels of MAP1B were increased in WT CGNs, whereas deletion of LAR, but not PTPσ, in CGNs largely eliminated the elevation of p-MAP1B levels (Fig. 4b,c). Together, the GSK-3β substrates APC and MAP1B mediate functions of PTPσ, but APC is not involved in LAR action, and MAP1B appears to mediate the action of LAR to a greater degree than that of PTPσ.

### Erk mediates actions of PTPσ and LAR by distinct downstream signaling pathways

Extracellular regulated kinases (Erks) are expressed in multiple cell types including neurons, and are essential for regulating protein synthesis/degradation and maintaining levels of proteins required for growth in response to many extracellular axon growth and guidance cues, including neurotrophins. Although it has not been determined whether Erk signaling mediates CSPG function, nerve growth factor (NGF) promoted growth of conditioning DRG neurons cultured on CSPGs^50^, suggesting a potential connection between the downstream activation of Erk by NGF and its neutralization of CSPG- mediated growth suppression. Therefore, we evaluated Erk activity by measuring phosphorylated p44/42 MAPK (Erk1/2) at Thr202/Tyr204. CSPG stimulation remarkably decreased levels of p-Erk in N2A cells transfected with either PTPσ or LAR (Fig. 4d). Importantly, CSPG application reduced levels of p- Erk in WT neurons, but deletion of either receptor alone failed to eliminate the inactivation effect on Erk signaling (Fig. 4e,f). These findings support that Erk signaling mediates actions of both RPTPs in neurons.

**Figure 5:**
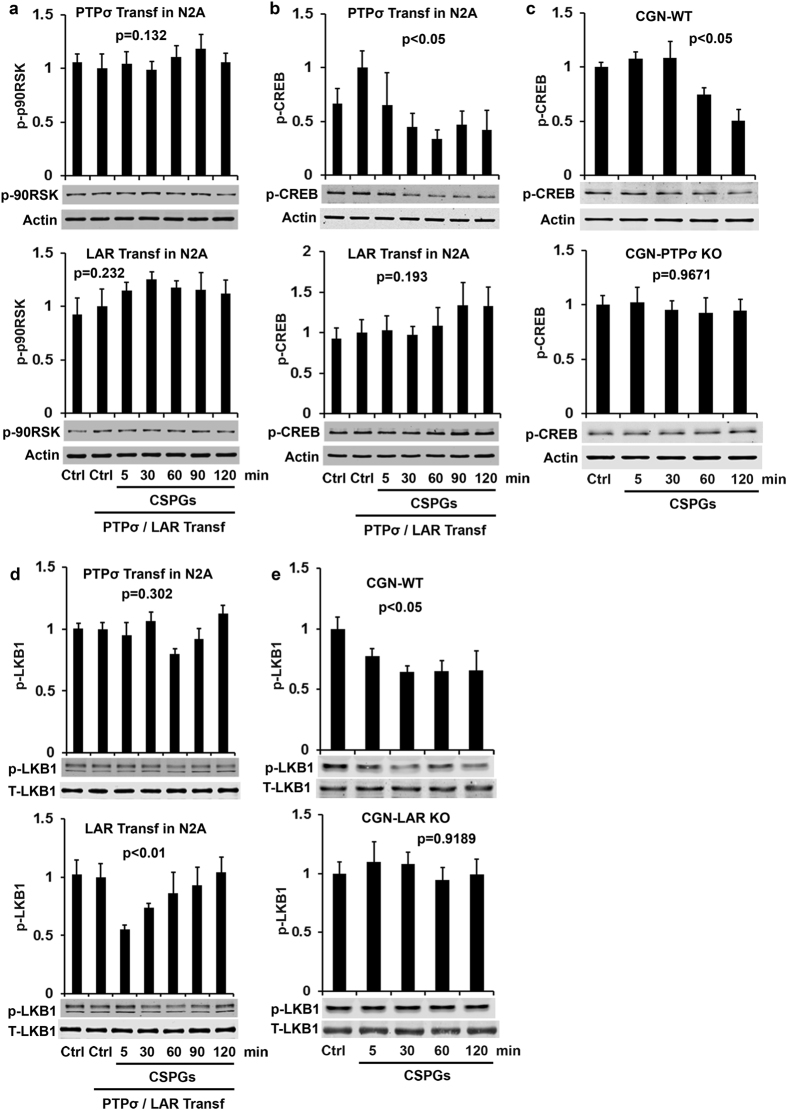
Effects of CSPGs on activities of p90RSK, CREB and LKB1 in N2A and CGN cultures. PTPσ/LAR transfected N2A cells or CGNs derived from PTPσ or LAR KO mice were treated with purified CSPGs (1.5 μg/ml) at several time points and the levels of phosphorylated p90RSK (p-p90RSK Thr573, active form), CREB (p-CREB Ser133, active form) and LKB1 (p-LKB1 Ser431, active form) in the supernatants of cell lysates were measured by Western blots. CSPGs did not alter levels of p-p90RSK in either PTPσ or LAR transfected N2A cells (**a**). CSPGs significantly decreased levels of p-CREB in PTPσ, not LAR, transfected N2A cells (**b**) and in PTPσ+/+, not PTPσ−/−, CGNs (**c**). In contrast, CSPGs stimulation decreased levels of p-LKB1 in LAR, not PTPσ, transfected N2A cells (**d**) and in LAR+/+, not LAR−/−, CGNs (**e**). The full-length blots are included in the Supplementary Information file.

The 90 kDa ribosomal S6 kinases (p90RSKs), also known as MAPK-activated protein kinase 1, are Ser/Thr kinases characterized by two non-identical functional kinase domains and a carboxy-terminal docking site for Erks^51^,^52^. The MAPK/Erk pathway activates p90RSKs by phosphorylating them at several sites within and outside of the RSK kinase domain, including Thr573. We thus measured p90RSK activity, but did not detect significant alteration of p-p90RSK (Thr573) levels in N2A cells overexpressing either PTPσ or LAR (Fig. 5a), suggesting that p90RSK plays a negligible role in mediating the functions of these two CSPG receptors.

Activation of Erk can enhance CREB phosphorylation at serine 133, thus increasing its activity as an upstream signal^53^,^54^. Because Erk mediates actions of both PTPσ and LAR (Fig. 4d–f), we examined the level of p-CREB (Ser133) and detected its reduction in N2A cells transfected with PTPσ, but not LAR (Fig. 5b). CSPGs also reduced the levels of p-CREB (Ser133) in WT CGNs and LAR deficiency abolished this effect (Fig. 5c). Thus, CREB modulates the interactions between CSPGs and PTPσ as a signal downstream of Erk.

**Figure 6:**
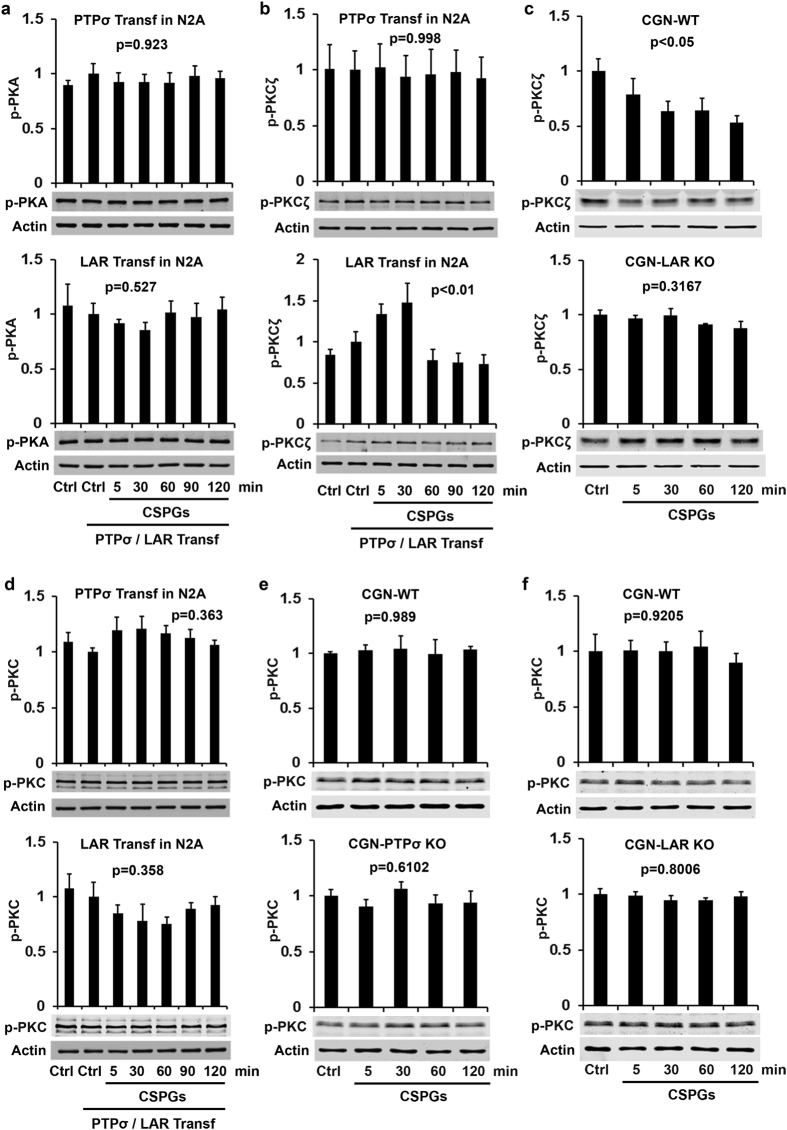
Effects of CSPGs on activities of PKA, PKCζ and PKC-pan in N2A cell or CGN cultures. PTPσ/LAR transfected N2A cells or CGN cultures derived from PTPσ or LAR KO mice were treated with purified CSPGs (1.5 μg/ml) at several time points and the levels of phosphorylated PKA (p-PKA Thr197, active form), PKCζ (p-PKCζ Thr410/403, active form) and PKC (p-PKC-pan Thr410, active form) in the supernatants of cell lysates were measured by Western blots. CSPGs did not alter levels of p-PKA in either PTPσ or LAR transfected N2A cells (**a**). CSPGs application increased levels of p-PKCζ in LAR, but not PTPσ, transfected N2A cells (**b**), but reduced levels of p-PKCζ in LAR+/+, not LAR−/−, CGNs (**c**). In contrast, CSPGs did not alter levels of p-PKC-pan in either PTPσ or LAR transfected N2A cells (**d**) and in PTPσ+/+, PTPσ−/−, LAR+/+ and LAR−/− CGNs (**e, f**). The full-length blots are included in the Supplementary Information file.

The serine/threonine kinase 11 (known as LKB1) and its downstream signaling target SAD/MARK regulate neuronal polarization and axon elongation during development^55^. Erk and RSK phosphorylate LKB1 at Ser325 and Ser428 (Ser431 in the mouse), which is critical for subsequent activation of AMP- activated protein kinase (AMPK)^56^. Because PTPσ and LAR activations suppress Erk activity, we examined the levels of p-LKB1 at Ser431, which is required for neuronal polarization^57^. PTPσ overexpression in N2A cells did not affect levels of p-LKB1 Ser431, but LAR activation due to CSPG stimulation decreased its levels (Fig. 5d). Moreover, CSPGs decreased levels of p-LKB1 in WT CGNs and LAR deletion eradicated this effect (Fig. 5e). The levels of p-LKB1 calibrated with either total LKB1 (Fig. 5d,e) or actin (Supplemental Fig. 2) exhibited the same changes (see Supplemental Table 1). Thus, CSPG-LAR (but not -PTPσ) interactions inactivate LKB1 signaling. On the other hand, cAMP- dependent PKA activates LKB1 by phosphorylating it at S431 and promotes axon differentiation during development^57^,^58^,^59^. We therefore measured the levels of p-PKA at Thr197, but detected only a trend toward slight reduction in LAR-overexpressing N2A cells after CSPG application (Fig. 6a, not significant), suggesting that PKA plays at most a minor role in mediating actions of the two RPTPs.

As a member of the atypical protein kinase C subfamily, PKCζ phosphorylates LKB1 at Ser-428/431 and mediates activation of AMPK in endothelial cells^60^,^61^. We measured p-PKCζ levels (Thr410/403) and detected its elevation in LAR- but not PTPσ-overexpressing cells following CSPG stimulation (Fig. 6b). CSPG treatment, however, reduced levels of p-PKCζ in WT but not LAR−/− CGNs (Fig. 6c). These experiments indicate that the PKCζ isoform contributes to LAR action upon CSPG application, probably by regulating LKB1 activity. Similarly, application of NG2, a non-lectican CSPG that is normally membrane-bound, regulated PKCζ activity in rat postnatal neurons and contributed to axon growth inhibition by NG2^62^. Because PKC appears to mediate actions of some axon growth inhibitors^63^, we also examined PKC activity by measuring levels of p-PKC-pan. In contrast, p-PKC was not altered in either LAR- or PTPσ-transfected cells following CSPG application (Fig. 6d). To confirm this result, we assessed its changes in CGN cultures and did not detect its alterations in either WT or LAR/PTPσ- deleted neurons (Fig. 6e,f). Therefore, PKC does not play a major role in CSPG-PTPσ/LAR interactions.

**Figure 7:**
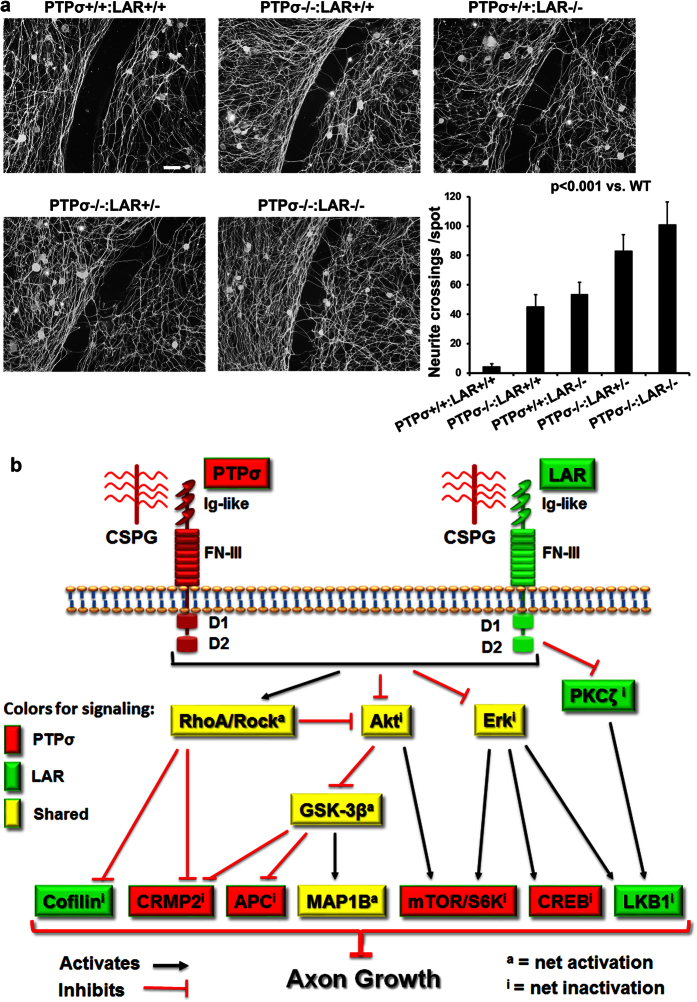
Additive effects for promoting neurite growth by deletion of both PTPσ or LAR receptors and schematic of downstream signaling pathways for two PTP receptors of CSPGs. Representative examples of neurite outgrowth on aggrecan spots show that a number of neurites crossed the aggrecan rim in DRGs derived from adult mice with one or two receptor deficiency, in contrast to no neurite crossing in DRGs derived from WT mice. The numbers of neurites that crossed the aggrecan rims were quantified from different groups (**a**). n = 17–33 spots from two separate experiments. Based on our findings in this study, we proposed a novel model of signaling pathways downstream of the two CSPG receptors (**b**). CSPGs inhibit neuronal growth by binding and activating two PTP receptors, PTPσ and LAR. Intracellularly, activation of PTPσ and LAR by CSPGs activate RhoA-Rock signaling and inactivate Akt and Erk pathways. However, the two receptors employ distinct pathways downstream of RhoA/Rock, Akt/GSK-3β and Erk signals to effect CSPGs’ inhibition of axon growth. Red-filled: signals conveyed by PTPσ. Green-filled: signals conveyed by LAR. Yellow-filled: shared signals by both PTPσ and LAR. Ig-like: immunoglobulin-like domains; FN-III: fibronectin Type III domains; D1: D1 domain; D2: D2 domain. S6K: S6 kinase.

Our experimental results with either N2A cells or primary neurons support that LAR and PTPσ share certain signaling pathways, but also employ distinct signals to covey CSPG effects on neurons. It was thus important to determine whether targeting both PTP receptors would have additive actions in overcoming CSPG-mediated inhibition of axon growth. CSPG-spot assay is a reliable method for measuring axon growth *in vitro* by counting the number of neurites that cross a gradient of CSPGs^15^,^64^, and we have demonstrated important roles for LAR and PTPσ in mediating the inhibition of axon growth by CSPGs with this and other models^16^,^19^. We now assessed whether deletion of both RPTPs would have synergistic actions in stimulating axon growth on a ring of high-concentration aggrecan. As shown in Fig. 7a, deleting either of these receptors enhanced neurite growth in adult DRG cultures on CSPGs, but DRGs derived from double KO mice exhibited a greater degree of axon growth compared to those from single KO animals. This finding suggests that the two RPTPs have synergistic effects in mediating CSPG suppression of axon growth *in vitro*.

## Discussion

To compare downstream signals of the two RPTPs, we conducted parallel experiments, measuring alterations of multiple signaling proteins in the lysates of PTPσ- or LAR-overexpressing N2A cells. These neuroblastoma-derived cultures possess the functional properties of neurons^26^ by differentiating into neurons, extending neurites and responding to various neuronal growth regulators^27^,^65^,^66^,^67^, including chondroitin sulfate, the representative sulfated glycosaminoglycan attached to the core proteins of CSPGs^68^. N2A cells are thus frequently used to study neuronal differentiation, neurite outgrowth, synaptogenesis, and neuronal signaling pathways. Importantly, we validated our findings in primary neuronal cultures derived from single or double KO mice. Previously, we and other groups had demonstrated that CSPGs activate RhoA/GSK-3β signaling and inactivate Akt signaling^21^,^22^,^34^,^35^,^69^, thus defining the roles of RhoA and Akt in mediating CSPG-LAR interactions^16^ in PC12 cells and mouse cerebellar neuron cultures. Therefore, the present results represent signaling pathways of general neuronal populations.

Based on the results of the present study and previous reports, we propose a simplified model of convergent and divergent pathways downstream of the two PTP CSPG receptors (Fig. 7b; see summary in Table 1). The interactions between CSPGs and LAR/PTPσ lead to activation of intracellular RhoA and inactivation of Akt and Erk signals. These 3 pathways are known to regulate growth of multiple cell types and elongation of axons. CSPG stimulation consistently activates RhoA and reduces phosphorylated Akt at Ser473 in LAR+/+, but not LAR−/−, cerebellar neurons^16^. Deletion of PTPσ activates MAPK and Akt signals in adult mouse retinal cells^70^. Downstream of RhoA, activation of Rock by PTPσ, but not by LAR action, inactivates the microtubule-interacting protein CRMP2 (Fig. 3), thus suppressing neuronal growth. Similarly, activation of Rock due to CSPG-PTPσ interactions appears to mediate outgrowth of oligodendrocyte processes and myelination^71^. In contrast, LAR-induced Rho activation is mediated by other signaling, including inactivation of the actin-binding protein cofilin (Fig. 2), which disassembles actin filaments and thus regulates dynamics of cytoskeleton during axon growth^32^,^34^,^72^,^73^.

PTPσ and LAR do not employ identical signaling to mediate CSPG actions, although both use the Akt/GSK-3β signaling pathway (Fig. 2). Activation of PTPσ by CSPGs alters activities of multiple microtubule-interacting proteins, including inactivation of CRMP2 and APC, as well as activation of MAP1B (Figs 3 and 4). LAR activation by CSPGs also activates MAP1B, but does not alter the activities of CRMP2 and APC. Moreover, the action of PTPσ, but not LAR, is mediated by ribosomal S6 kinase, one of the well-known downstream signals of the Akt/mTOR pathway. 4E-BP1, another well-known signal downstream of mTOR^39^, does not play a significant role in transmitting the actions of either PTPσ or LAR.

As a downstream signal for many neurotrophic factors^74^,^75^, Erk is linked to axon growth and regeneration^76^,^77^. CSPG and PTPσ/LAR interactions inactivate Erk signaling (Fig. 4), in addition to activating RhoA and inactivating Akt. Erk can phosphorylate and activate many substrates, some of which play important roles in controlling cell growth^78^. Among them, the ribosomal S6 kinases, including p70RSK (usually called S6 kinase) and p90RSK, are involved in neuronal signal transduction and growth^59^,^79^. We demonstrate that S6 kinase, but not p90RSK, contributes to the interactions between CSPGs and PTPσ (Figs 2 and 5) and that neither RSK conveys the action of LAR. Indeed, Erk phosphorylates S6 Kinase^80^ and its activation promotes axon regeneration after CNS injury^81^.

Moreover, activation of PTPσ, but not LAR, by CSPGs inactivated CREB by reducing its phosphorylation, suggesting that CSPG-PTPσ interactions inactivate Erk and subsequently reduce the activity of CREB, an important transcription factor for controlling neuronal growth^82^. Actually, Erk can phosphorylate CREB at serine 133 and thereby increase its activity^53^,^54^,^83^.

As a downstream signal of Erk, LKB1 appears to be critical for regulating axon elongation during development^55^. Our results measuring LKB1 Ser431 support a significant role for LKB1 in mediating the actions of LAR, but not PTPσ. Although LKB1 Ser325 is a direct phosphorylation target of Erk^56^, whether Erk phosphorylates LKB1 Ser431 directly or indirectly is unclear. We thus examined other signaling proteins that potentially link LAR to LKB1 and found that CSPG application increased PKCζ activity in LAR-transfected N2A cells. However, while CSPG stimulation reduced levels of p-PKCζ in WT CGNs, it did not do so in LAR−/− CGNs. These findings suggest that CSPG-LAR interactions inactivate PKCζ signaling in neurons. Why CSPGs activated this kinase in LAR-overexpressing N2A cells remains to be studied. Taken together, our results support the notion that CSPG-stimulated LAR activity suppresses LKB1 signaling by inactivating PKCζ in neurons. In contrast, our results did not support major roles for PKC or PKA in mediating the actions of either PTPσ or LAR.

Why PTPσ and LAR employ both convergent and divergent downstream signaling pathways remains unclear. It may relate to diversity in the ligands and substrates of RPTPs, indirect links along the different intracellular pathways and multiple interactions among the signals. Although CSPGs and Although HSPGs appear to be the substrates of PTPσ and LAR^15^,^16^,^84^,^85^, the physical ligands of these receptors are incompletely identified. N-cadherin, β-catenin, and p250GAP are the reported substrates of RPTPs^86^, but PTPσ and LAR may have other unidentified substrates. Stimulation of many transmembrane receptors can activate RhoA and inactivate Akt/Erk signals, but the interactions between different ligands and receptors may use diverse effectors to convey their functions. Various negative molecules for regulating cell growth, including CSPGs, myelin associated inhibitors and some repulsive guidance cues, can activate RhoA^6^,^87^, but they are unlikely to employ identical signaling pathways to suppress neuronal growth. Also, Rho signaling can regulate the activities of PTEN/Akt/mTOR proteins in some cell types, including macrophages, neutrophils^88^,^89^, and neurons^90^,^91^,^92^. The mechanisms by which RhoA is activated and Akt and Erk are inactivated by CSPG-RPTP interactions are still unknown.

To explain how PTPσ and LAR differ in their CSPG-signaling functions, we cannot exclude possible subtle roles of other signaling proteins that did not appear significantly altered by CSPG actions in the current experiments. For example, given the trend of their slight alterations (Fig. 3), 4E-BP1 may moderately mediate actions of PTPσ, while both 4E-BP1 and APC may modestly regulate LAR function. Other signals that were not studied here may play roles for mediating functions of the CSPG receptors. Expression of constitutively active Rac and Cdc42 GTPases enhanced neurite outgrowth on CSPG substrate^69^ and Rac appears to modulate PTPσ activity through p250GAP and subsequent cytoskeleton dynamics^93^. Interrupting LAR function with a systemically delivered small peptide appears to activate Trk receptors and focal adhesion-associated protein kinase FAK^94^,^95^. Thus, it will be important to further study potential regulating functions of these and other signals.

We detected a crucial role of atypical PKCζ, but not the conventional PKC, in regulating CSPG-LAR interactions (Fig. 6). In contrast, a previous study indicated that CSPG application enhanced activity of conventional PKC by increasing levels of p-PKC-pan^63^. It is likely that CSPGs activate PKC-pan by PTPσ-/LAR-independent pathways. Not only myelin-associated growth inhibitors, but also CSPGs signal axon growth inhibition by binding to Nogo receptors^17^,^63^. Therefore, it will be interesting to determine whether CSPGs activate PKC by interacting with NgRs, especially NgR1 and 3. Because PKC is required for Rho activation by myelin-associated growth inhibitors^63^, future studies also should be aimed at determining whether activation of PKCζ by CSPG-LAR interactions has a similar effect on Rho activity.

In this study, we examined 15 signaling proteins that potentially act as the downstream pathways mediating the interactions between CSPGs and two PTP receptors (Table 1) and used actin as a loading control for some of them. We have confirmed the reliability of using actin as a loading control by performing comparison experiments and demonstrating that the alterations of signaling proteins calibrated with either total signaling proteins or actin are basically identical in both types of cells (Supplemental Table 1). Consistently, actin has been proven to be a reliable loading control for multiple cultured cells and is widely used for studies with Western blot assay^96^. Because p-cofilin is a regulator of actin dynamics, we further confirmed its alterations in LAR-transfected N2A cells and WT CGNs by calibration with both total cofilin and actin.

In identifying the signaling pathways of the two CSPG receptors, we primarily employed one-way ANOVA to determine whether CSPG stimulation induces statistically significant differences at several time points. The overall alterations of the signaling proteins in N2A cells and CGN cultures matched well. Because the properties of N2A cells may not be identical to those of primary CGNs, the extents and latencies of the signaling changes might not be the same. For example, p-S6 showed a greater degree of reduction at more delayed time points in CGNs than in PTPσ-transfected N2A cells (Fig. 2e,f). However, we identified changes of the signaling proteins in N2A cells and confirmed them with primary cultures of postnatal CGNs. Therefore, our proposed model in Fig. 7b should apply to CNS neurons.

## Conclusions

On the basis of the present results and previous studies, we summarize convergent and divergent pathways that mediate the axon growth-inhibitory effects of CSPG-RPTP interactions in Fig. 7b. This diagram highlights some areas where more studies are required to resolve apparent discrepancies. Both LAR and PTPσ activate RhoA/Rock and inhibit Akt and Erk, but the mechanisms by which these effects are produced are not known. Although Rho/Rock inhibits both Cofilin and CRMP2, only LAR inhibits Cofilin and only PTPσ inhibits CRMP2. Similarly, mTOR/S6K and CREB are activated by Akt and Erk, but only PTPσ inhibited S6K and CREB signals. It thus is very important to further study intermediate signals downstream of PTPσ and LAR and their potential links with the identified pathways, including RhoA, Akt and Erk. Moreover, it will be interesting to determine whether specific interventions of the identified pathways (such as blocking or/and activating them) can alter the CSPG receptors-mediated responses and axon growth ability on CSPG substrates. Because we evaluated a large number of downstream signaling proteins in this study, it is beyond the scope of this paper to further investigate their significance in axon regeneration over CSPG inhibitors.

Transgenic or pharmacological inhibition of either PTPσ or LAR induces significant regrowth of several projection fiber tracts after SCI, including serotonergic, corticospinal and sensory fibers, and improves functional recovery^16^,^18^,^19^. Given widespread expression of these RPTPs in adult CNS^15^,^16^,^97^, their suppression should promote growth of other tracts, such as the rubrospinal, reticulospinal and propriospinal axons. Because PTPσ and LAR conveyed CSPG signaling by both convergent and divergent pathways, blocking both RPTPs simultaneously should produce additive effects in promoting axon regeneration. Consistent with this, deletion of both PTPσ and LAR enhanced adult neuronal axon growth on CSPGs *in vitro* to a greater degree than deletion of either receptor alone (Fig. 7a). Moreover, suppressing CSPG actions, when combined with other strategies that address different mechanisms of axon growth failure, including regulating inflammatory responses and activities of other signaling pathways (*e.g.*, PTEN, SOCS3, STAT3 and c-myc) or transcription factors (such as KLFs)^38^,^98^,^99^,^100^, may induce more robust axon regeneration than any single therapy alone. Combining axon regenerative strategies with task-specific rehabilitative training should further promote rewiring of appropriate neuronal circuits, reinforcing functionally meaningful synaptic reconnections and enhancing neuroplasticity after CNS injuries.

## Materials and Methods

### Ethical approval

All experimental protocols were approved by the Institutional Animal Care & Use Committee at Temple University and the methods were carried out in accordance with the relevant guidelines and regulations.

### Sources of compounds

Antibodies against the following proteins were used: mouse mAb phospho-Akt (Ser473, 587F11), rabbit mAb phospho-S6 ribosomal protein (Ser235/236), rabbit mAb phospho-Erk1/2 (p44/42), rabbit pAb phospho-CRMP2 (Thr514), rabbit pAb phospho-cofilin (Ser3), rabbit mAb phospho-4E- BP1 (eukaryotic initiation factor 4E-binding protein 1, Thr37/46, 236B4), rabbit mAb phospho-CREB (Ser133, 87G3), rabbit mAb phospho-PKA C (Thr197, D45D3), rabbit pAb phospho-90 kDa ribosomal S6 kinase (p90RSK, Thr573), rabbit pAb phospho-PKCζ/λ (Thr410/403), rabbit mAb phospho-PKC (pan) (zeta Thr410, 190D10), rabbit mAb phospho-LKB1 (Ser428, C67A3) (all from Cell Signaling Technology), rabbit pAb phospho-MAP1B (Thr1265, Millipore), rabbit pAb phospho-APC (Ser2054, Abcam), mouse anti-actin clone C4 (MP Biomedicals) and mouse mAb against RhoA (sc-418; from Santa Cruz Biotechnology). The major proteins employed include rhotekin binding domain beads (binds active Rho protein, RT02-A, Cytoskeleton, Inc), a mixture of purified CSPGs (containing neurocan, versican phosphacan and aggrecan, Millipore), laminin (Sigma) and several protease inhibitors (see below, Sigma,). A plasmid with the human LAR sequence was provided by Dr. Morgan Sheng and amplified in our lab^16^,^101^. A plasmid with the human PTPσ sequence was generated and amplified in our lab.

### N2A cell cultures and sample preparations

N2A, a mouse neural crest-derived cell line, possesses neuronal properties^26^ and is widely used to study neuronal differentiation, axonal growth and signaling pathways^65^,^66^,^67^,^102^,^103^. N2A cells were grown on poly-L-lysine-coated 35 mm dishes in Dulbecco’s Modified Eagle’s Medium/Nutrient Mixture F-12 Ham supplemented with 10% fetal bovine serum, 2 mM glutamine, 100 μg/ml penicillin and 100 μg/ml streptomycin. One day after growth, cells were transfected with control plasmid, WT LAR or PTPσ plasmid, using Xfect transfection reagents (Clontech Laboratories). Two days after transfection, cells were stimulated with vehicle or purified CSPGs (1.5 μg/ml) for 5–120 minutes as indicated in Figs 1, 2, 3, 4, 5 and 6. Following 3 washes with ice-cold PBS, cells were prepared in 300 μl cold lysis buffer supplemented with protease inhibitors (1 mM phenylmethylsulfonyl fluoride, 2 mM orthovanadate, 10 μg/ml leupeptin and 10 μg/ml aprotinin). Samples were clarified by centrifugation at 15,000 g for 10 min at 4 °C and total protein was determined with Bio-Rad DC protein assay reagents. Samples containing the same amount of total protein were then aliquoted into multiple tubes and stored at −80 °C for biochemical assays.

### PTPσ and LAR knockout mice and cerebellar granular neuron cultures

PTPσ and LAR knockout mice were provided by Drs. Michel Tremblay^104^ and Frank Longo^105^, respectively. PTPσ and LAR +/− littermate crosses were used to generate +/+, +/− and −/− littermates. Genotyping was conducted by PCR (for PTPσ KO) or reverse transcription-PCR following the protocols from Dr. Longo’s Lab (for LAR KO)^105^. Homozygous and heterozygous PTPσ and LAR mutant mice are viable and grossly normal in appearance. Primary cerebellar granular neuron cultures were prepared from the cerebelli of postnatal 7–9 day mice derived from PTPσ+/+, PTPσ−/−, LAR+/+ or LAR−/− mice. After trypsin digestion, cells were dissociated and grown in culture medium (DMEM/F12 containing 10% fetal bovine serum, 2 mM glutamine, 100 μg/ml penicillin and 100 μg/ml streptomycin, 25 mM KCl and 25 mM glucose) for 24 hrs at 37 °C inside of 6-well plates. Before cell plating, the wells were prepared by coating with poly-L-lysine (100 μg/ml) and incubating with 10 μg/ml laminin for 2 hrs at 37 °C. NGF was not applied to neuronal cultures for neurite outgrowth assays. CGNs were then treated with purified CSPGs at indicated time points.

### Active RhoA assay

Supernatant samples from N2A or CGN cultures (in 35 mm dishes) were prepared as above. After quantification of total proteins in lysates, using Bio-Rad DC protein assay reagents, a sample containing the same amount of proteins from each dish was incubated with rhotekin binding domain coupled beads (45 μg/sample) for 50 min at 4 °C. GTP-bound RhoA and total RhoA in cell lysates were detected by Western blot using a mouse monoclonal antibody against RhoA^106^. Proteins were transferred to nitrocellulose membranes and bands were visualized with enhanced chemiluminescence reagents as above. For blot densitometry, the images of active RhoA bands were captured with a Bio-Rad Gel Doc XR documentation system and the band density was determined using Quantity One software^21^. The intensity of an individual band was calculated by subtraction of the value of background in the same lane from which the band was measured, and then calibrated against the intensity of the total RhoA band.

### Western blot assay

Expression changes of each studied signaling protein were determined with Western blots in N2A cells and CGNs after CSPG treatment. The supernatants of cell lysates containing the same amount of total proteins were loaded onto Tris-Glycine gels and transferred to nitrocellulose membranes. The membranes were blocked with 5% blotting grade milk (BIO-RAD), blotted with primary antibodies described above, and then incubated with appropriate secondary antibodies conjugated to IRDye 800CW (LI-COR) or IRDye 680RD (LI-COR). To determine the levels of loading control proteins, membranes were probed with anti-total signaling proteins and/or actin using separate membranes with the same amount of total loading proteins. Visualization and quantification were carried out with the LI-COR Odyssey^®^ scanner and Odyssey^®^ imaging software. For the Western blotting assays, at least three separate experiments were performed and the representative blots are shown in the figures.

### Dorsal root ganglion cultures and CSPG gradient assay for neurite outgrowth

DRGs were harvested from various genotypes of mice (6–7 weeks old, Fig. 7a) and incubated in collagenase (100 U/ml, Worthington Biochemical Corporation, Lakewood, NJ) and then with collagenase plus 0.25% trypsin/EDTA. After wash with culture medium, dissociated DRG neurons were plated onto plastic coverslips and grown in culture medium (DMEM/F12 mixture plus 10% fetal bovine serum, 2 mM glutamine, 100 μg/ml penicillin and 100 μg/ml streptomycin) for 5 days at 37 °C^21^,^35^. Before cell plating, coverslips (15 mm) coated with poly-L-lysine were spotted with a 2-μl solution of aggrecan (600 μg/ml) and laminin (10 μg/ml) in HBSS-CMF (2 spots/coverslip). After complete dryness of the spots, coverslips were incubated with laminin (10 μg/ml) in HBSS-CMF at 37 °C for 2 hr. Five days after plating, DRG cultures were fixed in 4% paraformaldehyde in PBS for 30 min. After several rinses with PBS, coverslips were incubated in blocking solution (5% normal goat serum + 0.2% BSA in 0.1% Triton X-100 PBS) for 1 hr at room temperature and then incubated with anti-βIII-tubulin (1:400; Covance) overnight at 4 °C. After rinses several times with PBS, DRG cultures were incubated in appropriate secondary antibody (ThermoFisher Scientific) for 1 hr at RT. The number of βIII-tubulin positive axons crossing spot rim (visualized by CS56 staining) were counted from each spot. Multiple coverslips were employed for each group of individual experiments.

### Statistical analysis

SigmaPlot software was used for statistical analysis. Data in graphs are shown as means ± SEM. The comparisons between multiple groups were analyzed with a repeated measures ANOVA. The experiments comparing a single determination of means between two independent groups, including the active RhoA and neurite growth assay, were analyzed with Student’s *t*-test. Differences between groups with *p* < 0.05 were considered significant (*p < 0.05, **p < 0.01). Quantification data for each signaling protein were collected from 3–5 separate experiments.

## Additional Information

**How to cite this article**: Ohtake, Y. *et al.* Two PTP receptors mediate CSPG inhibition by convergent and divergent signaling pathways in neurons. *Sci. Rep.* **6**, 37152; doi: 10.1038/srep37152 (2016).

**Publisher’s note**: Springer Nature remains neutral with regard to jurisdictional claims in published maps and institutional affiliations.

## Supplementary Material

Supplementary Information

## Figures and Tables

**Table 1:** Comparison of signaling pathways downstream of PTPσ and LAR.

Proteins	Fig.	N2A cells PTPσ-Transf	CGNs		N2A cells LAR-Transf	CGNs		Activity
			PTPσ+/+	PTPσ−/−		LAR+/+	LAR−/−	
Active RhoA	1	↑p < 0.05	↑p < 0.05	↔p = 0.2664	↑p < 0.05	↑(Fisher *et al.*)	↔(Fisher *et al.*)	↑by both
p-Cofilin ^i^	2	↔p = 0.234	ND	ND	↑p < 0.01	↑p < 0.05	↔p = 0.4838	↓by LAR
p-Akt	2	↓p < 0.01	↓p < 0.01	↔p = 0.879	↓p < 0.01	↓(Fisher *et al.*)	↔(Fisher *et al.*)	↓by both
p-S6	2	↓p < 0.01	↓p < 0.05	↔p = 0.8677	↔p = 0.147	ND	ND	↓by PTPσ
p-4E-BP1 ^i^	3	Trend ↓P = 0.119	ND	ND	Trend ↓p = 0.154	ND	ND	Trend ↑by both
p-CRMP2 ^i^	3	↑p < 0.01	↑p < 0.05	↔p = 0.9466	↔p = 0.163	ND	ND	↓by PTPσ
p-APC	3	↓p < 0.01	↓p < 0.05	↔p = 0.9387	Trend ↓P = 0.146	ND	ND	↓by PTPσ
p-MAP1B	4	↑P < 0.01	↑p < 0.05	↑p < 0.05	↑p < 0.01	↑p < 0.05	↔p = 0.1922	↑by both
p-Erk	4	↓p < 0.01	↓p < 0.05	↓p < 0.05	↓p < 0.01	↓p < 0.05	↓p < 0.05	↓by both
p-p90RSK	5	↔p = 0.132	ND	ND	↔p = 0.232	ND	ND	↔by both
p-CREB	5	↓P < 0.05	↓p < 0.05	↔p = 0.9671	↔p = 0.193	ND	ND	↓by PTPσ
p-LKB1	5	↔p = 0.302	ND	ND	↓p < 0.01	↓p < 0.05	↔p = 0.9189	↓by LAR
p-PKA	6	↔p = 0.923	ND	ND	↔p = 0.527	ND	ND	↔by both
p-PKCζ	6	↔p = 0.998	ND	ND	↑p < 0.01	↓p < 0.05	↔p = 0.3167	↑or ↓by LAR
p-PKC(pan)	6	↔p = 0.563	↔p = 0.989	↔p = 0.6102	↔p = 0.358	↔p = 0.9205	↔p = 0.8006	↔by both

Note: All phosphorylated proteins indicate their active form except for p-CRMP2, p-Cofilin, and p-4E-BP1, which are marked with ^i^. ND: not determined.
