# Supplementary material for: Two PTP receptors mediate CSPG inhibition by convergent and divergent signaling pathways in neurons
Source: Sci Rep. 2016 Nov 16;6:37152. doi: 10.1038/srep37152 (PMC5111048; doi:10.1038/srep37152)
Supplement: Supplementary Information [file srep37152-s1.pdf]

Two PTP receptors mediate CSPG inhibition by convergent and divergent signaling pathways in neurons

Yosuke Ohtake, Daniella Wong, P. M. Abdul-Muneer, Michael E. Selzer, and Shuxin Li

Supplemental Table 1. Comparison of the results with two loading proteins: actin and total signaling protein.

Proteins	Fig.	N2A cells	CGNs		N2A cells	CGNs		Activity
		PTPσ-Transf	PTPσ +/+	PTPσ -/-	LAR-Transf	LAR +/+	LAR -/-	
**p-Cofilin/actin**	S1	↔ $p = 0.195$	ND	ND	↑ $p < 0.01$	↑ $p < 0.05$	↔ $p = 0.9886$	↓ by LAR
**p-Cofilin/T-Cofilin**	2	↔ $p = 0.234$	ND	ND	↑ $p < 0.01$	↑ $p < 0.05$	↔ $p = 0.4838$	↓ by LAR
**p-Akt/actin**	S1	↓ $p < 0.01$	↓ $p < 0.01$	↔ $p = 0.974$	↓ $p < 0.01$	ND	ND	↓ by both
**p-Akt/T-Akt**	2	↓ $p < 0.01$	↓ $p < 0.01$	↔ $p = 0.879$	↓ $p < 0.01$	↓ (Fisher et al)	↔ (Fisher et al)	↓ by both
**p-LKB1/actin**	S2	↔ $p = 0.261$	ND	ND	↓ $p < 0.01$	↓ $p < 0.05$	↔ $p = 0.7875$	↓ by LAR
**p-LKB1/ T-LKB1**	5	↔ $p = 0.302$	ND	ND	↓ $p < 0.01$	↓ $p < 0.05$	↔ $p = 0.9189$	↓ by LAR

Note: ND: not determined.

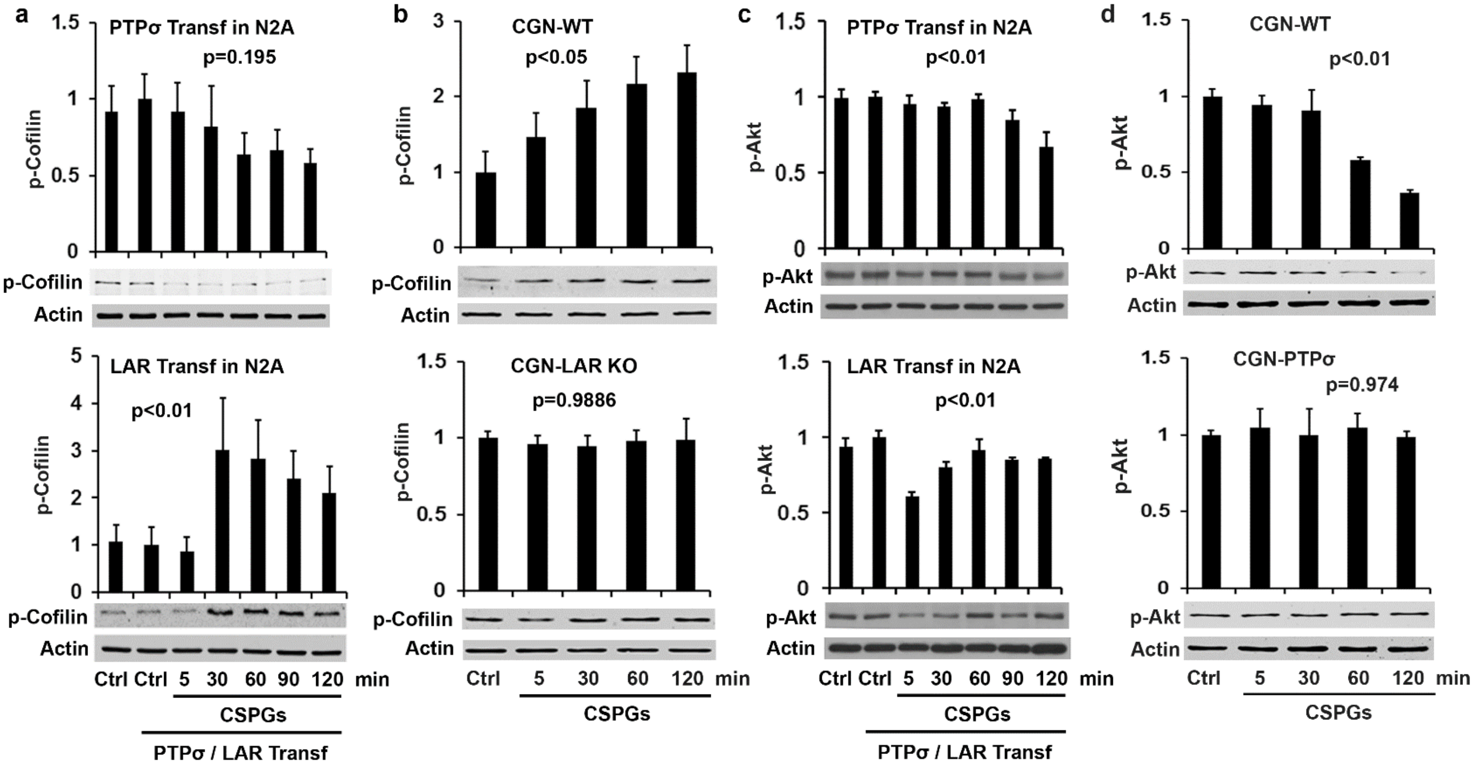

Supplemental figure 1 *Effect of CSPG on activities of cofilin and Akt in N2A cells or CGNs measured with actin as a loading protein.* PTPσ or LAR transfected N2A cells or CGNs derived from PTPσ or LAR KO mice were treated with purified CSPGs (1.5 µg/ml) for different lengths of time, and the levels of phosphorylated Cofilin (p-Cofilin Ser3, inactive form, **a** and **b**) and Akt (p-Akt s473, active form, **c** and **d**) in the cell lysates were measured by Western blots. The levels of p-Cofilin and p-Akt were calibrated with levels of actin in the same samples, instead of with total Cofilin and Akt as in Fig. 2. Use of actin as a loading control indicated the same alterations of these signaling proteins as in Fig. 2.

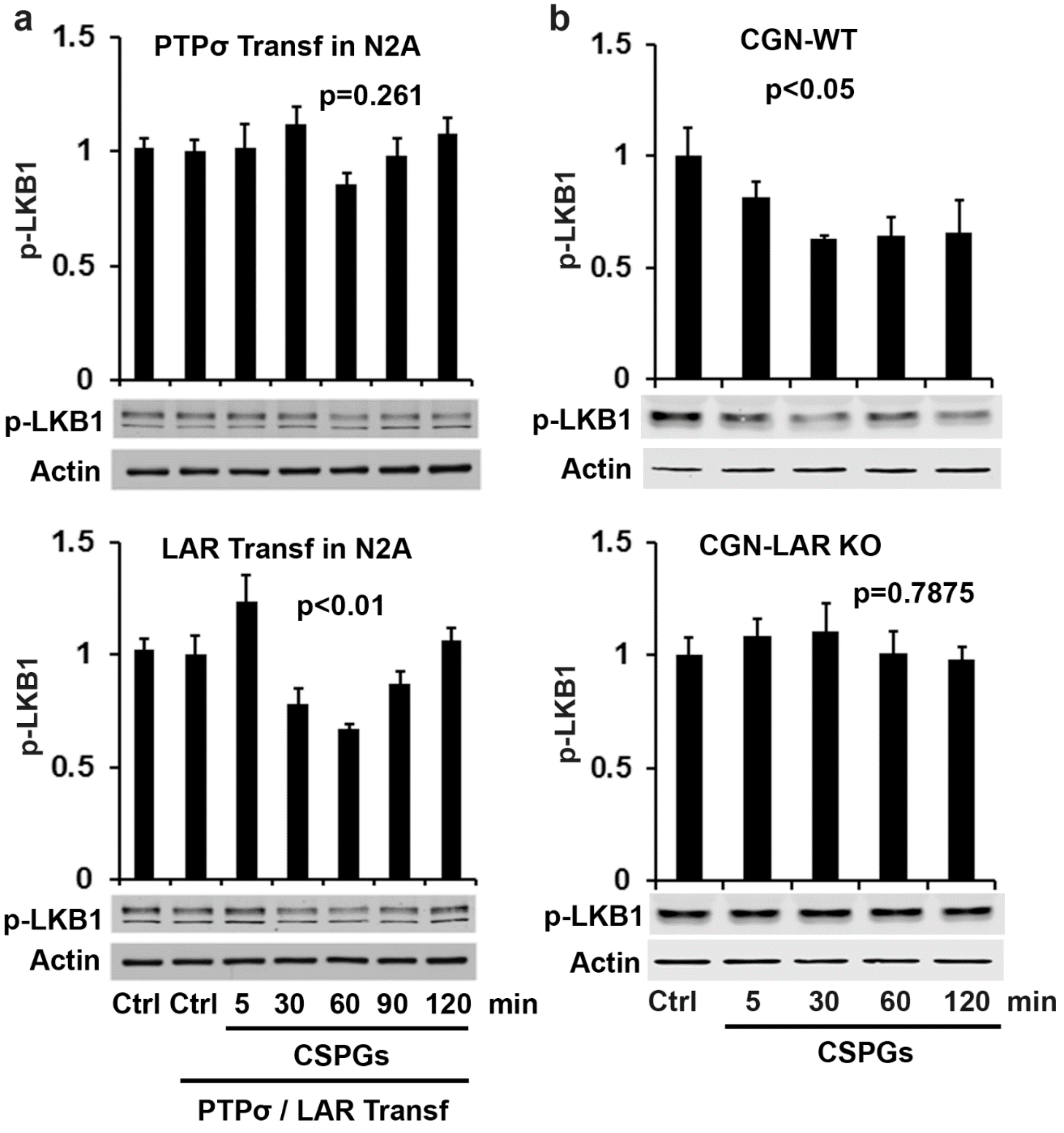

Supplemental figure 2 *Effect of CSPG application on activities of LKB1 in N2A cells or CGNs, measured with actin as a loading protein.* PTPσ/LAR transfected N2A cells or CGNs derived from LAR KO mice were treated with purified CSPGs (1.5 µg/ml) for several lengths of time and the levels of phosphorylated LKB1 (p-LKB1 Ser431, active form) in the cell lysates were measured by Western blots. The levels of p-LKB1 were calibrated with levels of actin in the same samples, instead of with total LKB1 as in Fig. 5. Use of actin as a loading control consistently showed that CSPGs stimulation decreased levels of p-LKB1 in LAR, not PTPσ, transfected N2A cells (**a**), and in LAR+/+, not LAR-/-, CGNs (**b**).

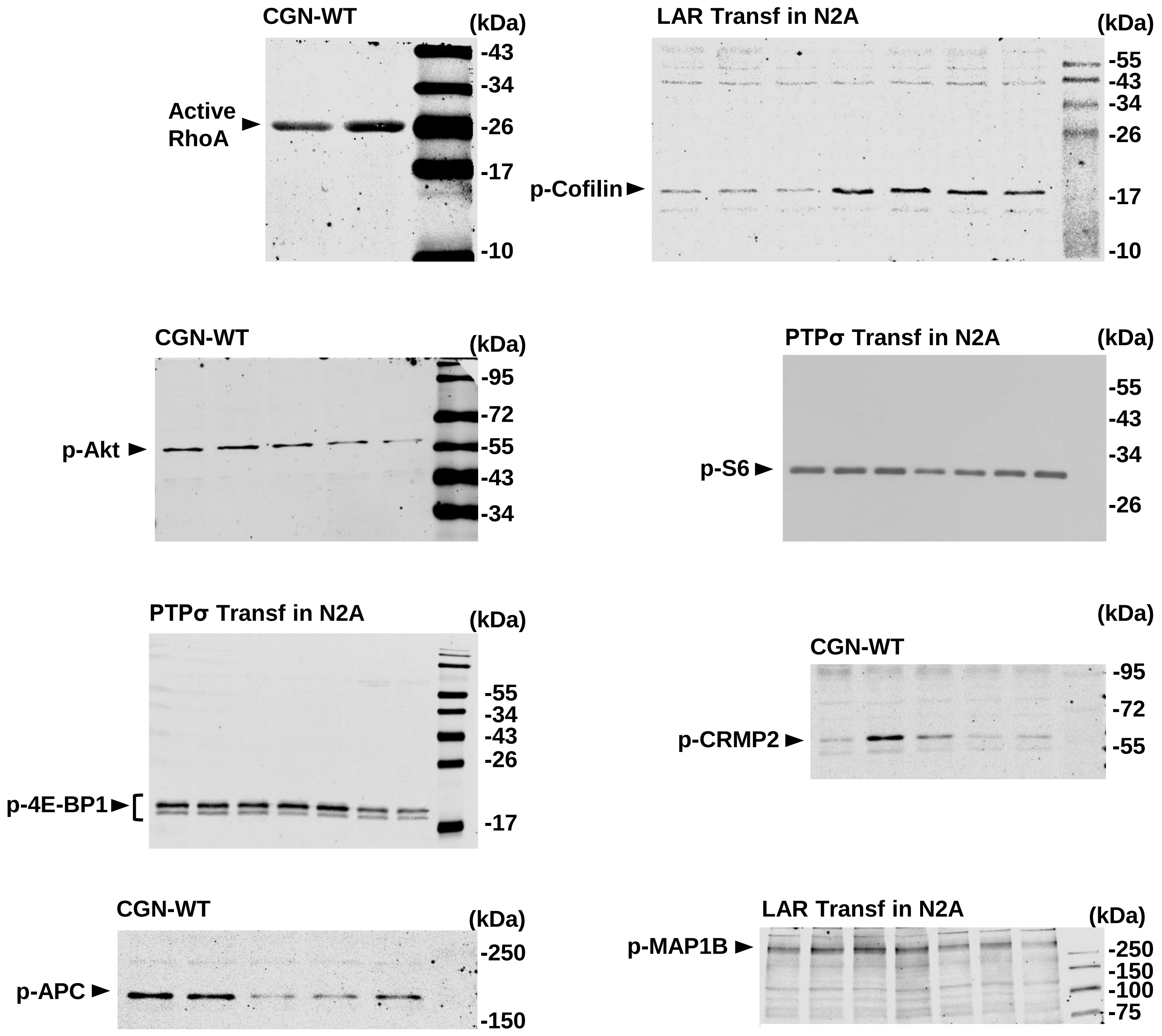

Supplemental figure 3 *The full-length blots are shown for active RhoA, p-Cofilin, p-Akt, p-S6, p-4E-BP, p-CRMP2, p-APC, and p-MAP1B.*

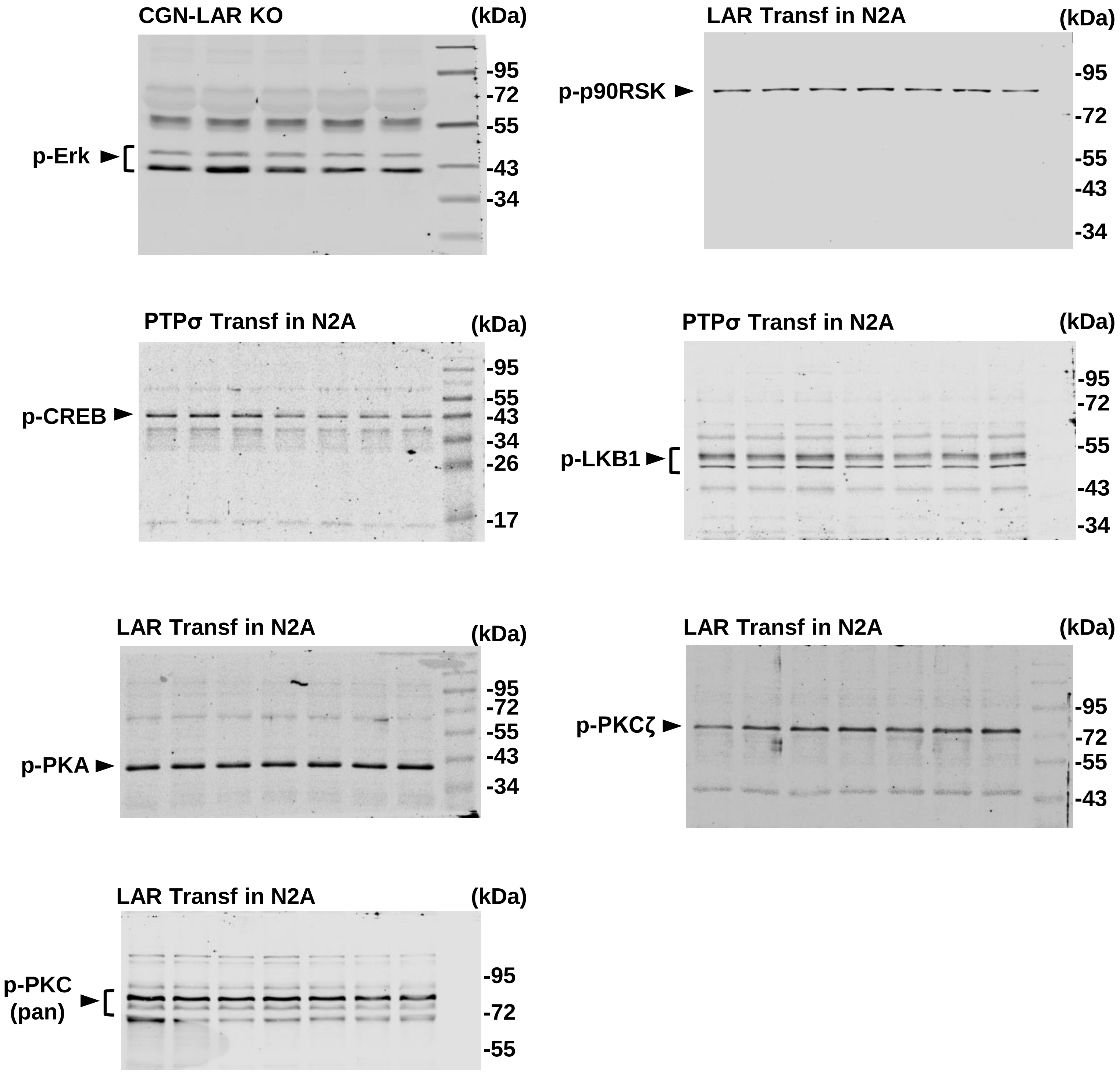

Supplemental figure 4 *The full-length blots are shown for p-Erk, p-p90RSK, p-CREB, p-LKB1, p-PKA, p-PKCζ, and p-PKC(pan).*
